# Aerosol particle emission increases exponentially above moderate exercise intensity resulting in superemission during maximal exercise

**DOI:** 10.1073/pnas.2202521119

**Published:** 2022-05-23

**Authors:** Benedikt Mutsch, Marie Heiber, Felix Grätz, Rainer Hain, Martin Schönfelder, Stephanie Kaps, Daniela Schranner, Christian J. Kähler, Henning Wackerhage

**Affiliations:** ^a^Institute of Fluid Mechanics and Aerodynamics, Department of Aerospace Engineering, Universität der Bundeswehr München, 85577 Neubiberg, Germany;; ^b^Professorship of Exercise Biology, Department of Sport and Helth Sciences, Technische Universität München, 80809 Munich, Germany;; ^c^Institute of Computational Biology, Helmholtz Zentrum München, 85764 Neuherberg, Germany

**Keywords:** aerosol particle concentration, aerosol particle emission, physical activity, exercise, SARS-CoV-2

## Abstract

Many airborne pathogens such as severe acute respiratory syndrome coronavirus 2 (SARS-CoV-2) are transmitted indoors via aerosol particles. During exercise, pulmonary ventilation can increase over 10-fold, and therefore, exercisers will exhale a greater volume of aerosol-containing air. However, we currently do not know how exercise affects the concentration of aerosol particles in exhaled air and the overall emission of aerosol particles. Consequently, we developed a method to measure in parallel the concentration of aerosol particles in expired air, pulmonary ventilation, and aerosol particle emission at rest and during a graded exercise test to exhaustion. We used this method to test eight women and eight men in a descriptive study. We found that the aerosol particle concentration in expired air increased significantly from 56 ± 53 particles/liter at rest to 633 ± 422 particles/liter at maximal intensity. Aerosol particle emission per subject increased significantly by a factor of 132 from 580 ± 489 particles/min at rest to a super emission of 76,200 ± 48,000 particles/min during maximal exercise. There were no sex differences in aerosol particle emission, but endurance-training subjects emitted significantly more aerosol particles during maximal exercise than untrained subjects. Overall, aerosol particle emission increased moderately up to an exercise intensity of ∼2 W/kg and exponentially thereafter. Together, these data might partly explain superspreader events especially during high-intensity group exercise indoors and suggest that strong infection prevention measures are needed especially during exercise at an intensity that exceeds ∼2 W/kg. Investigations of influencing factors like airway and whole-body hydration status during exercise on aerosol particle generation are needed.

## Significance

Airborne transmission of severe acute respiratory syndrome coronavirus 2 (SARS-CoV-2) or other pathogens is probably increased during indoor exercise, but data on the emission of aerosol particles by an exercising individual are lacking. Here, we report that aerosol particle emission increases on average 132-fold from 580 ± 489 particles/min at rest to 76,200 ± 48,000 particles/min during maximal exercise. Aerosol particle emission increases moderately up to an exercise intensity of ≈2 W/kg and exponentially at higher exercise intensities. These data not only explain SARS-CoV-2 transmissions during indoor group exercise but also can be used to design better targeted mitigation measures for physical activity indoors such as physical education in school, dance events during weddings, or high-intensity gym classes such as spinning.

Coronavirus disease 19 (COVID-19) is an infectious respiratory disease that can kill especially elderly individuals (1). COVID-19 is caused by variants of severe acute respiratory syndrome coronavirus 2 (SARS-CoV-2) that is more infectious and more deadly than, e.g., influenza (2). In addition to serious respiratory disease and death, SARS-CoV-2 can damage many organs (3) and can cause long COVID in >10% of cases, where symptoms persist for an average of 328 d (4). By January 2022, more than 300 million people were infected with SARS-CoV-2 and nearly 5.5 million people have lost their lives due to COVID-19 (5), making the COVID-19 pandemic the most severe pandemic since the flu pandemic of 1918 to 1920.

In winter 2021/2022, the situation has changed compared to the first COVID-19 winter of 2020/2021. This may be explained by the following: the administration of 9 billion vaccine doses worldwide (5), natural SARS-CoV-2 infections have immunized others, and the less pathogenic Omicron variant is currently dominant in many countries. As a result, fewer SARS-CoV-2-infected individuals became hospitalized or died of COVID-19 in winter 2021/2022 than in winter 2020/2021 (5). Despite that, many countries still impose mitigation measures to avoid severe COVID-19 in nonvaccinated individuals and very high caseloads.

Physical activity and recreational and competitive sports not only are an important part of our culture and leisure activities but also prevent many diseases (6). Exercise is also an effective treatment for at least 26 major diseases (7). COVID-19 mitigation measures have, however, changed and often reduced physical activity. For example, Canadian and UK citizens were 30 to 40% less physically active (8), which had an effect on physical and mental health.

Indoor group exercise in small, poorly ventilated rooms promotes infection with SARS-CoV-2 (9, 10). SARS-CoV-2 and other airborne pathogens are transmitted via aerosol particles with a diameter up to a few hundred micrometers and droplets of a few hundred micrometers or larger (11). The larger droplets are carriers of pathogens during infections that occur when two persons are up to ∼1.5 m to each other as they quickly drop to the ground. In contrast, small aerosol particles float in the air, can carry pathogens such as SARS-CoV-2, and can mediate airborne infections. Direct contact between persons or via shared objects can also cause infections but this is less likely (12). Several studies have shown that the concentration of aerosol particles in the expired air varies greatly in the population. For example, roughly 20% of individuals emit more than 156 particles per liter of exhaled air. These individuals have been termed “superemitters” (13). The number of aerosol particles emitted by a person per unit of time varies and increases when that person talks, coughs, sings (14, 15), or is physically active (16–20). It has also been shown that the number of emitted aerosol particles is influenced by the airway hydration status (20, 21). Dehydration of the airways can be caused by exercise and increased ventilation and both could lead to a higher number of emitted aerosol particles. During exercise, ventilation (i.e., the air inhaled and exhaled by a person) increases from ∼5 to 15 liter/min at rest to over 100 liter/min in untrained individuals (22) and can reach 200 liters/min in highly trained rowers (23). Finally, it is reported that mildly SARS-CoV-2-infected individuals expire higher aerosol particle numbers than noninfected individuals (20). Together, this suggests that exercising SARS-CoV-2-infected individuals will “blow out” more SARS-CoV-2 into a room and that exercising, noninfected individuals will inhale more SARS-CoV-2-contaminated aerosol particles, when compared to rest.

Current research on airborne pathogen transmission is limited by the fact that most studies only report the concentration of aerosol particles in expired air (13, 14, 18, 20, 24–26) or in a room (16–19, 23) but not the emission of aerosol particles by one individual. Since the latter is required for assessing the risk of infection, we developed a method to measure both the concentration and emission of aerosol particles from rest up to maximal exercise. The aim of this study was to use this method to answer the following three research questions:

1) What is the ventilation, aerosol particle concentration per liter of exhaled air, and aerosol particle emission at rest and during a graded cycle ergometry test to exhaustion?
2) Do women and men as well as untrained and endurance-trained subjects differ in their aerosol particle emission?
3) Does the highly variable aerosol particle emission at rest predict aerosol particle emission at different exercise intensities?

## Results

**Fig. 1.**
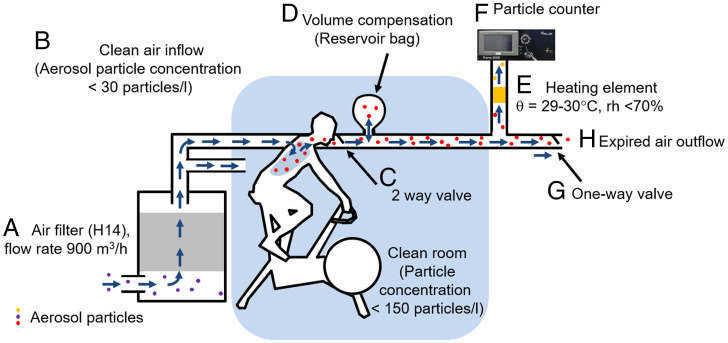
Schematic illustration of the experimental set up designed to measure ventilation, aerosol particle concentration, and aerosol particle emission at a wide range of ventilation from rest to maximal exercise. Ambient air was first filtered (*A*) to generate air that is nearly free of aerosol particles. The subject then inhaled the (*B*) filtered, clean air through a silicone face mask that covered mouth and nose (not shown). The silicone mask was (*C*) connected to a two-way valve so that only exhaled air entered the outflow. A plastic bag acted as a buffer/reservoir (*D*). A pump diverted ∼5 liter/min of the exhaled air through first (*E*) a heated tube to eliminate condensation and then to the (*F*) Palas Promo 3000 particle counter. This counter uses a Welas 2300 sensor for particle detection. The remaining air was released into the environment through a separate tube and a one-way valve (*G*) so that ambient air could not enter the system. The experiment was conducted in a clean room to further reduce the risk of aerosol particle contamination.

**Fig. 2.**
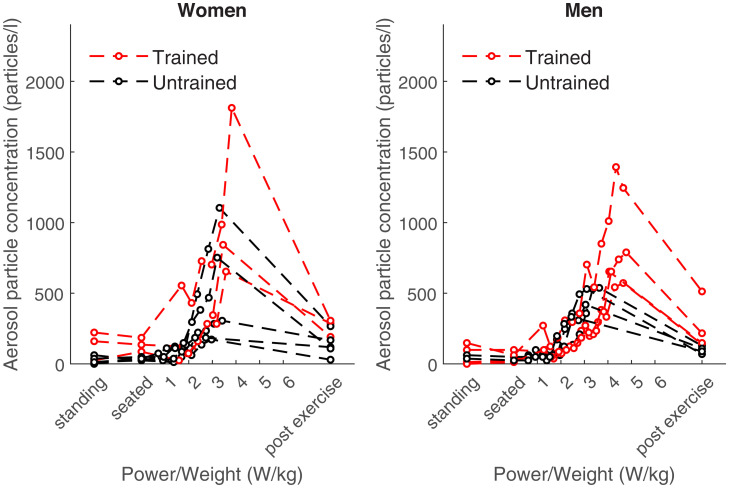
Aerosol particle concentration in the exhaled air at rest and at different exercise intensities in women (*n* = 8, *Left*) and men (*n* =8, *Right*). Shown are standing and seated (on ergometer) values in the order of the test procedure.

We first designed a method for measuring aerosol particle emission at rest and during a graded exercise test to exhaustion in humans, which is schematically illustrated in Fig. 1. We then used this experimental set up to measure ventilation, aerosol particle concentration, and aerosol particle emission, which is the product of ventilation and the concentration of aerosol particles in the exhaled air. We found that the aerosol particle concentration increased on average in all subjects significantly over 10-fold from 56 ± 54 particles/liter at rest to 633 ± 422 particles/liter (range, 103 to 1,551 particles/liter; *P* < 0.001) during maximal exercise (Fig. 2). There was no significant difference between women and men (*P* > 0.05), but the aerosol particle concentration of untrained subjects during maximal exercise was 509 ± 222 particles/liter (range, 184 to 813 particles/liter) that was significantly lower than that of endurance-trained subjects who emitted 877 ± 525 particles/liter (range, 345 to 1,812 particles/liter) during maximal exercise (*P* = 0.025). The concentration of particles per liter of expired air reached maxima of >1,000 particles/liter in two women and one man. Particle size distribution did not differ between biological sexes and did not change significantly during exercise (*SI Appendix*, Fig. S6; mean particle size, 0.46 ± 0.05 µm).

**Fig. 3.**
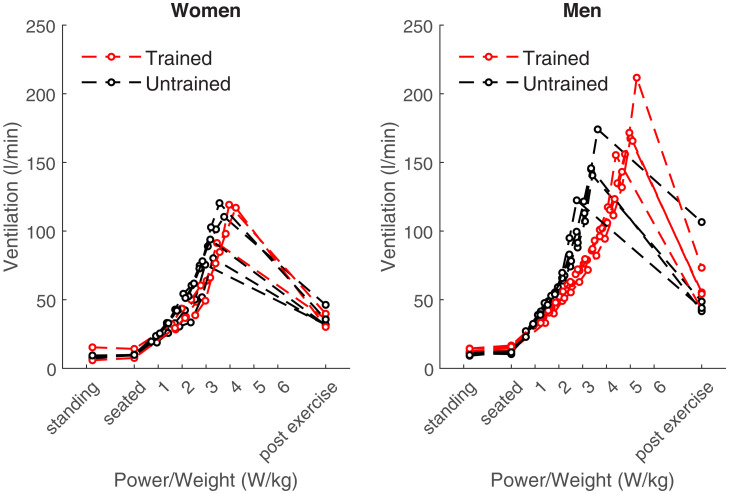
Ventilation at rest and at different exercise intensities in women (*n* = 8, *Left*) and men (*n* = 8, *Right*).

The increase of ventilation during exercise is well known. In our subjects, ventilation increased significantly from 9 ± 2 liter/min at rest to exercise maxima of 101 ± 18 liter/min (range, 75 to 120 liter/min) in women and from 13 ± 2 liter/min at rest to exercise maxima of 160 ± 27 liter/min (range, 122 to 211 liter/min) in men (*P* < 0.001), respectively (Fig. 3). On average, men ventilated significantly more than women during maximal exercise (*P* < 0.001). Moreover, trained subjects ventilated 147 ± 40 liter/min during maximal exercise than untrained subjects who reached 118 ± 32 liter/min. This difference was not statistically significant due to the large interindividual variability of maximal ventilation (*P* = 0.186).

**Fig. 4.**
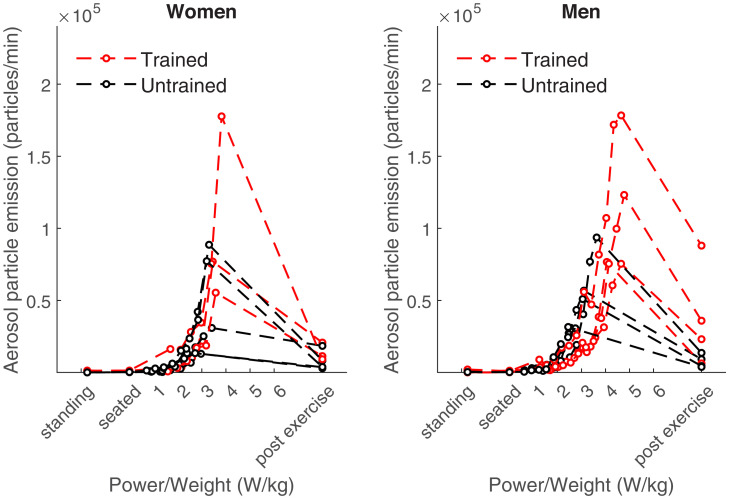
Aerosol particle emission at rest and at different exercise intensities in women (*n* = 8, *Left*) and men (*n* = 8, *Right*). Standing and seated (on ergometer) values are in the order of the test procedure.

In our experiments, aerosol particle emission increased on average 132-fold from 579 ± 489 particles/min at rest to 76,200 ± 48,000 particles/min during maximal exercise (*P* < 0.001) (Fig. 4). On average, men exhaled 29% more particles during maximal exercise than women that was not statistically significant (*P* = 0.804). Endurance-trained subjects exhaled 85% more particles during maximal exercise than untrained subjects that was a significant difference (*P* = 0.02). Especially from an exercise intensity of 2 W/kg (e.g., 150 Watt for a 75-kg individual) upward, mean aerosol particle emission exceeded 10,000 particles/min.

**Fig. 5.**
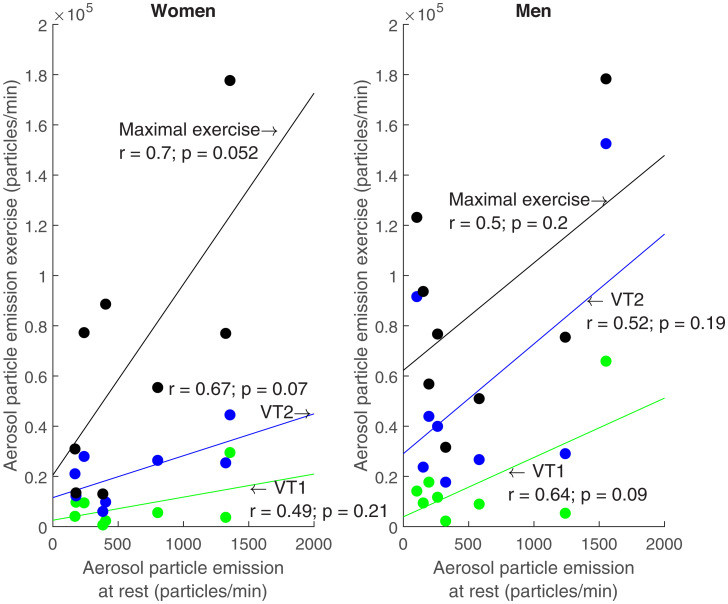
Aerosol particle emission at rest in comparison to three specific exercise intensities (VT1 and VT2 and maximal exercise) for all women (*n* = 8, *Left*) and men (*n* = 8, *Right*).

Next, we asked the following question: Does a high resting aerosol particle emission predict a high aerosol particle emission during exercise? To address this question, we correlated the resting aerosol particle emission with aerosol particle emissions at different exercise intensities (ventilatory thresholds 1 [VT1] and 2 [VT2] and maximal exercise) for each participant and plotted these data in Fig. 5. We found that the one female and male subject with the highest aerosol particle emission at rest also had the highest aerosol particle emission during maximal exercise. Overall, aerosol particle emission at rest correlated moderately (27) with aerosol particle emission at maximal intensity (*r* = 0.58, *P* = 0.02). However, we found no significant correlation for men (*r* = 0.50, *P* = 0.2) and women (*r* = 0.70, *P* = 0.052) when rest was compared to aerosol particle emission at the first (VT1) and second ventilatory threshold (VT2) or maximal exercise intensity (Fig. 5). This suggests that aerosol particle emission at rest is not a reliable biomarker for aerosol particle emission during exercise.

## Discussion

The first main result of this descriptive study is that aerosol particle emission increases on average 132-fold from 580 ± 489 particles/min at rest to a superemission of 76,200 ± 48,000 particles/min during maximal exercise in healthy, young women and men. We also found that aerosol particle emission increases moderately up to an exercise intensity of ∼2 W/kg and exponentially at higher exercise intensities. This finding can be used to design improved mitigation strategies for indoor group exercise. Third, aerosol particle emission at rest is only moderately correlated with aerosol particle emission during exercise.

The goal of this study was to measure aerosol particle emission over the whole range of human ventilation from ∼5 to 15 liter/min at rest to over ∼200 liter/min during maximal exercise. To be able to do this, we needed to developed an experimental set up that detects the concentration of particles in a partial flow of exhaled air without correction (24–26) that reduces the errors that can be caused by uncontrolled inflow of particle-free air (13, 14, 27, 28). By measuring aerosol particle concentration and ventilation in one individual, we were able to calculate aerosol particle emission that is a more direct measure of the risk of pathogen transmission by aerosol particles by one individual than the aerosol particle concentration in exhaled air or in room air (16–19, 25).

Despite using a different experimental set up, our resting aerosol particle concentrations are within the range of aerosol concentrations reported by other studies. Specifically, we measured 56 ± 54 particles per liter of exhaled air at rest. This compares to published mean values of ∼25 particles/liter (14), <100 particles/liter (25), 230 particles/liter (range, 18 to 1,000 particles/liter [28]), and 250 particles/liter (24) at rest. Our data are at the lower end of the published range. This could be due to the fact that we sought to minimize the aerosol content of the inspired air by filtering it (Fig. 1).

We found that the mean aerosol particle emission increased 132-fold from rest to maximal exercise. In comparison, Wilso, et al. (26) counted 58 times more aerosol particles in expired air during submaximal exercise of 70% of the maximal heart rate than at rest. Furthermore, George et al. (20) showed in healthy nonsmokers (*n* = 20) that 30 min of strenuous exercise led to an increase in the expired aerosol particle concentration from 58.8 particles/liter to 220.6 particles/liter in the postexercise recovery phase. Notably, the participants showed high interindividual variation. Explaining this unexpected increase of the concentration of aerosol particles during exercise is a challenge for future studies. Possible factors include changes in the velocity and type (i.e., turbulent versus laminar) of gas flow, changes of the composition of the liquids that line the airways, and changes in the hydration status of the airways (20, 21). Thus, besides exercise intensity, the airway dehydration that was potentially caused by the endurance exercise and higher ventilation rates could have contributed to the increased aerosol particle emission. In our study, the concentration of aerosol particles was 633 ± 422 particles/liter that is greater than the increase of the exhaled aerosol particle concentration reported for loud speech that was 320 particles/liter (14). However, when a subject inhales and exhales rapidly, then the particle concentration can reach 2,800 particles/liter (24). Taken together, while it is difficult to compare aerosol particle emission with aerosol particle concentration, these data suggest that speaking and a deeper inspiration and expiration increases the particle concentration in expired gas. During intensive exercise, this is exacerbated by increases of ventilation by a factor 10 or more.

We found that endurance-trained athletes emitted 85% more particles during maximal exercise than untrained subjects (*P* = 0.02). This is intuitive as endurance-trained individuals also ventilate more aerosol-containing air into a room during maximal exercise. In this study, endurance-trained subjects ventilated on average 25% more than untrained subjects during maximal exercise that was not significant due to a large interindividual variation. This difference only partially explains the variation in particle emission between untrained and endurance-trained subjects. The other factors that contribute to the 85% difference in aerosol emission between trained and untrained subjects are unknown.

To answer the question whether the aerosol emission at rest predicts high aerosol emission during exercise, we performed correlation analyses. This revealed that aerosol particle emission at rest moderately predicted aerosol particle emission at the VT1 and VT2 and at maximal exercise (Fig. 5). Somewhat similar, Asadi et al. (14) reported that breathing aerosol high emitters were not necessarily speech high emitters and vice versa. They attributed this to different mechanisms of aerosol particle generation, namely, aerosol particle production by the lung or through speech generation. Our measurements show that even without speaking, aerosol particle generation while breathing at rest and during exercise seems to be based on different mechanisms. This is similar to the findings of Almstrand et al. (28), who showed that an increase in airway opening results in higher aerosol particle formation and emission.

Our data have important implications for infection control during indoor group exercise. Previous studies have identified indoor group exercise as a situation where SARS-CoV-2 outbreaks can occur (9, 10, 29). Our data show that aerosol emission increases moderately up to ∼2 W/kg and exponentially at higher intensities (Fig. 4 and *SI Appendix*, Fig. S7). We therefore recommend for exercise up to an intensity of ∼2 W/kg to consider keeping a distance of >1.5 m between exercisers, a high exchange or filtering rate of room air, and limited time spent in the exercise room (e.g., 45 to 90 min). As Buonanno et al. (30) proposed that the majority of outbreaks were caused by the combination of one superspreader in a small room with poor ventilation, we recommend additional measures to reduce the risk of infection during indoor group exercise above 2 W/kg when superemission occurs. This could include 15-min airing breaks in between classes, pre-exercise infection testing of participants, safety shields in between exercisers, mobile air filters (19, 31), and the wearing of masks even during exercise (26, 32–34).

Our study has limitations. The aim of our and similar experiments is to quantify the risk of airborne transmission of SARS-CoV-2 or other pathogens by an individual at rest and during exercise. While aerosol particle emission is a major contributor to that risk, it is not the only one, as infection risk also depends on the concentration of the pathogen in the exhaled aerosol particles. So, ideally, others and we would have measured aerosol particle emission and the SARS-CoV-2 titer in aerosol condensates of infected, COVID-19 patients. However, we were unable to perform such an experiment safely. Moreover, high-intensity exercise experiments with COVID-19 patients are arguably unethical, as exercise may increase the risk of complications such as myocarditis (3). Findings in SARS-CoV-2-infected monkeys (13) and SARS-CoV-2-infected humans (20) suggest that SARS-CoV-2-infected organisms emit more aerosol than healthy controls. Thus the measured increases of aerosol particle emission during exercise in our study are probably lower limits and some SARS-CoV-2-infected individuals may emit even more aerosol particles during exercise.

A second limitation is that the aerosol particle concentration at rest and exercise intensities below 2 W/kg is low and variable so that the relative measurement uncertainty is greater than at high or maximal exercise intensity (*SI Appendix*, Figs. S4 and S5). We reduced the resultant measurement variability by averaging over a period of 4 min during each stage of the experiment. This measurement duration is in the range of 1- to 5-min measurement intervals reported in the literature (18, 19, 24, 26, 35). The duration is shorter than that reported by Milton et al. (36) who measured indirectly the expired aerosol particle concentrations for 30 min at rest and also shorter than the 30 min of Helgeson et al. (17) who measured the concentration of aerosol particles in room air during physical exercise with an increasing intensity (3 × 10 min light, hard, very hard).

A further limitation is that possible changes of the hydration status of the subjects were not assessed, as the hydration status can affect aerosol particle emission (20, 21). Subjects were allowed to drink water ad libitum in the 3 h before the test to avoid pretest dehydration. As exercise leads to general dehydration (36) and can thus also induce dehydration of the airways, a proper record of the pre- and posthydration status of the subjects is needed for the in-depth interpretation of aerosol particle emission data.

In summary, we report that aerosol particle emission increased by a factor of 132 from rest to maximal exercise with no significant difference between women and men but between untrained and endurance-trained subjects during maximal exercise. Aerosol particle emission increases moderately up to an exercise intensity of 2 W/kg and exponentially at higher exercise intensities. This information should be used to develop more data-based mitigation measures for indoor group exercise.

## Materials and Methods

### Study Design and Subjects.

We conducted an observational, monocentric human cohort study with the main aim to continuously measure ventilation, the concentration of aerosol particles in the expired air, and aerosol particle emission at rest and during a graded cycle exercise test to exhaustion. All measurements and procedures were approved by the medical ethical committee of the Technical University Munich. Prior to each test, participants and staff were tested for SARS-CoV-2 with an antigen test. Eligible participants were healthy males and females aged 18 to 40 y (see *SI Appendix*, Table S1). We recruited all subjects (*n* = 16) from local sports clubs, the faculty of Sport and Exercise Science of the Technical University Munich, and the Universität der Bundeswehr München. Participants were included or excluded based on their pulmonary, cardiovascular health and needed to be fully vaccinated against SARS-CoV-2. Participants were excluded if they were smokers, ill, suffered from asthma, or had recently experienced respiratory problems. Based on their V̇O_2_max (i.e., a key measure of cardiorespiratory fitness), we classified all male and female subjects as either endurance trained (V̇O_2_max, ≥55 mL/min/kg for men; V̇O_2_max, ≥45 mL/min/kg for women; *n* = 8) or as untrained (V̇O_2_max, <55 mL/min/kg for men and <45 mL/min/kg for women; *n* = 8). All subjects signed an informed consent before all tests and experiments.

### Two Cardiopulmonary Exercise Tests.

Each subject performed two graded cycle cardiopulmonary exercise tests with an identical test protocol on two separate days following standard guidelines for such tests. Before each of the two tests, subjects were requested to avoid intensive training up to 24 h before the test and were asked to eat a regular/normal diet. They were also asked not to drink coffee, black tea, and other beverages containing caffeine before each test. Subjects fasted during the last 3 h before each test but could drink water ad libitum to remain hydrated. The exact amount of water intake prior to the exercise test and the hydration status were not controlled.

During both tests, subjects cycled on a bicycle ergometer (Excalibur Sport). During the first test, we continuously measured ventilation (V’E in liters/min), breathing frequency (BF in liters/min), and tidal volume (VT in liters) with a calibrated spiroergometry device (Metalyzer; Cortex Medical). From the data of the first test, we later determined the two ventilatory thresholds VT1 and VT2 and the V̇O_2_max. During the second test, we continuously measured aerosol particle concentrations as described below (see *Aerosol particle measurement*). The mean relative humidity was 39 ± 8% (minimum 30%) and mean temperature was 22 ± 3 °C during the experiments.

During the first cardiopulmonary exercise test, we started by measuring lung function and recorded forced expiratory volume in the first second (FEV1 in liter/s) and the forced vital capacity (FVC in liters). After that, both tests were identical and comprised the following parts:

1) Start of continuous measurement (test 1, ventilation and other cadiopulmonary data; test 2, aerosol particle concentration).
2) Standing rest for 4 min.
3) Rest while sitting on the cycle ergometer for 4 min.
4) Graded cycle exercise test as follows:
  a) Untrained subjects started by cycling 50 W for 4 min. After that, load was increased by 25 W all 4 min until subjective exhaustion.
b) Endurance-trained subjects started by cycling 100 W for 4 min. After that, load was increased by 25 W all 4 min until subjective exhaustion.
5) After the end of the exercise, subjects recovered for 5 min while sitting on the cycle ergometer.
6) End of continuous measurement.

### Aerosol Particle Measurement.

The aim of the second test was to measure the concentration of aerosol particles in the expired air using a near-identical test protocol as during the first test where we measured ventilation and other cardiopulmonary data. If the maximal exercise intensity in the second exercise test was lower than during the first test, then we recorded the lower maximal exercise intensity of both tests as the overall maximal intensity reached. During the second test, we continuously measured the expired aerosol particle concentration with an optical particle counter (Palas Promo 3000 particle spectrometer using a Welas 2300 sensor, Palas). This device allows the determination of the aerosol particle size distribution and aerosol particle concentration (particles/liter) simultaneously. We quantified dried particles of 0.2 up to 10 μm in diameter.

To ensure that subjects inhaled air with no/few aerosol particles, we filtered the inspired air with a H14 filter that was connected to a face mask via tubing. The expired air that left the face mask was directed via a two-way valve to the outflow system. From the outflow, we diverted a gas flow of 5 liters/min to the Palas Promo 3000 particle spectrometer to measure the concentration of aerosol particles. To avoid condensation in the sensor, the supply line to the sensor was heated to 29 to 30 °C and brought to a relative humidity of ∼70%. Nevertheless, some condensation occurred in the mask, the blowback flap housings, and the duct up to the heating element. This may reduce the concentration of larger water droplets and thereby also alter particle size distribution. The particle size measurement may be associated with a small bias error since the refractive index of the aerosol particles is not precisely known. We used the refractive index of latex particles as is common practice. To further reduce the risk of aerosol contamination by leakage, we carried out the experiment in an air space/tent that was ventilated with filtered air.

### Data Processing.

All data were extracted and processed with Matlab (version R2021b). Mean values were calculated for each interval. In the analysis, we only included steps of the graded exercise test with a minimum duration of 60 s. For resting values, standing and seated steps were averaged. This was done to reduce measurement error due to the 8-min interval time.

### Statistical Analysis.

All data were analyzed using PRISM (GraphPad Prism 9.0.0(121)). Normality and sphericity were tested and accounted for. Either two-way ANOVA, ANOVA, or Kruskal–Wallis test was used.

## Supplementary Material

Supplementary File

## Data Availability

All study data are included in the article and/or supporting information.
