# Supplementary material for: Aerosol particle emission increases exponentially above moderate exercise intensity resulting in superemission during maximal exercise
Source: Proc Natl Acad Sci U S A. 2022 May 23;119(22):e2202521119. doi: 10.1073/pnas.2202521119 (PMC9295808; doi:10.1073/pnas.2202521119)
Supplement: Supplementary File [file pnas.2202521119.sapp.pdf]

PNAS
www.pnas.org

Supplementary Information for
Aerosol particle emission increases exponentially above moderate exercise intensity resulting in superemission during maximal exercise

Benedikt Mutsch[1]*, Marie Heiber[2]*, Felix Grätz[2], Rainer Hain[1], Martin Schönfelder[2], Stephanie Kaps[2], Daniela Schranner[2,3], Christian J. Kähler[1]*& Henning Wackerhage[2]*

1 Universität der Bundeswehr München, Institute of Fluid Mechanics and Aerodynamics, 2 Technische Universität München, Associate Professorship of Exercise Biology, 3 Helmholtz Zentrum München, Institute of Computational Biology

Email: benedikt.mutsch@unibw.de

This PDF file includes:

Supplementary text
Figures S1 to S7
Tables S1

Other supplementary materials for this manuscript include the following:

Dataset and statistics

Supplementary Information Text

In the following sections we provide detailed information on methods and data acquisition, as well as data on ventilation, particle size and aerosol concentration in a constant load setting.

Detailed methods

A Recruitment and Design

Subject were recruited during May to December 2021 from local sports clubs, gyms, and several institutions of the TUM and Universität der Bundeswehr München using information flyers and social media channels. Participants had to be aged between 18 and 40 years and being free of cardiovascular, pneumological, neurological, neoplastic, metabolic, allergic or any other disease that would preclude a maximal exercise test. Further exclusion criteria were being a smoker and drinking alcohol on a regular basis. For women, being pregnant was another exclusion criterion. Subjects were asked for their training status in regard to their VO2max based on their last exercise test performed or based on the VO2max assumption from commercial sport watches. According to this subject were assigned to the trained or untrained group (see Table 1). As an infection with SARS-CoV-2 increases the number of emitted aerosols (Edwards et al., 2021) only subjects fully vaccinated (2 or 3 times) against or recovered completely from a SARS-CoV-2 infection were included in the study. Furthermore, we performed a SARS-CoV-2 Antigene Test before each testing / on each assessment day. Altogether, two different subject groups of each four males and females were recruited.

All parameters were assessed on two separate assessment days (Fig. S1). The first day included a medical check-up and a graded exercise CPET until volitional exhaustion. On the second day, following 48 h to 14 days later, aerosol emission was measured using the identical graded exercise protocol.

B Medical Check and CPET

On the day before the experiments, subjects had to refrain from intensive exercise and alcohol and stick to their usual sleep and nutrition routine regarding timepoints and amount. Before the test, the subjects should be fasting for about 3 h and drink water only. Both endurance groups were advised to refrain from caffeine 24 hours before the tests, the resistance trained athletes should refrain from caffeine on the day of measurements. After arrival at the laboratory, all staff and subjects were tested with rapid antigen SARS-CoV-2 test kits (Rapid SARS-CoV-2 antigen-test, Xiamen Boson Biotech™, China) to minimize the risk of infection for all involved persons as well as the influence of a pre- or asymptomatic SARS-CoV-2 infection on the amount of emitted aerosols (Edwards et al., 2021).

C Graded exercise CPET

Subjects showed up at the lab between 7-10:00 am in the morning. After we recorded their height and weight, they lay down in supine position. Electro cardiography (ECG) electrodes (Kendall™, Germany) were placed left and right below the clavicula and left and right below the iliac crest as reference electrodes for the resting ECG. Electrode C1 was placed in the fourth intercostal space at the right edge of the sternum, C2 at the corresponding left edge. C4 was placed in the fifth intercostal space at the left medio clavicular line and C3 on the fifth rib between C2 and C4. C5 was placed left on the frontal axillary line at the height of C4, C6 on the corresponding site and height at the mid axillary line. After connecting the leads to the corresponding channels, ECG were generated using a Bluetooth ECG device (Custo Cardio 200™; Customed, Germany). Subjects lay silent without any movement for five minutes while the resting ECG was recorded. Afterwards, still in supine position, we measured the blood pressure manually with a blood pressure cuff (KI, Bosch & Sohn™, Germany) and a stethoscope (Littmann™ Classic 3, 3M, USA) according to Riva-Rocci.

Doctors then checked the subjects for inclusion / exclusion criteria and pre-existing conditions. They also asked for regular medication intake, medical history, regular exercise, allergies, and smoking and drinking habits. They then checked head and neck, lung, and heart during a physical examination. If no exclusion condition was met, subjects were given the approval for the maximal exercise test. We then re-placed the reference electrodes on the insertion of the deltoid muscle left and right side and on the back on the height of the ninth rib left and right side and the ECG device was then reconnected for the according channels.

Subjects adjusted a bicycle ergometer (Excalibur Sport; Lode™, Netherlands) according to their individual preferences regarding saddle and bar vertical and horizontal position. Subjects were allowed to use pedals with straps and normal sport shoes or clipless cycling shoes (SPD; Shimano™, Japan).

While subjects adjusted the ergometer, we calibrated a pre-warmed spiroergometry device (Metalyzer; Cortex Medical™, Germany) according to the manufacturer's manual. It is an open-circuit, valveless device, measuring respiratory gases and ventilation breath-by-breath. The flow sensor was calibrated with a volume flow of two to four l/s in- and expiration and the gas sensor with fresh ambient air before each measurement and with calibration gas (15.00 O_2 / 5.00 CO_2) if required by the software. We then attached the spiro-ergometry mask (7450 V2; Hans Rudolph, USA) with the flow sensor to the subjects faces and secured it with straps. Ventilation parameters were recorded breath-by-breath and reported for each second.

We performed a maximal flow volume curve test with each subject before each cardiopulmonary exercise test. Subjects stood upright with their face away from the screen and were asked to breath in a normal rhythm. After the third normal inspiration, subjects exhaled deeply, followed by a rapid and full inhalation. Subjects then had to exhale fast (high force) and maximally, until a plateau in the volume trend could be seen, though at least six seconds. Based on the flow-volume curve, inspiratory capacity (IC), forced vital capacity (FVC), and the forced expiratory volume in the first second (FEV1) were determined.

After finishing the bike set-up and a successful flow-volume-maneuver, we re-connected the ECG-device to the software. We started the protocol with the subject being in a standing position. We used a step protocol with four-minute steps, first standing, then sitting and then exercising with 25 watts (W) increment per step. Endurance-untrained athletes started with 50 W, endurance-trained athletes with 100 W until volitional exhaustion. After the maximal intensity was reached, subjects remained in a seated position for another five minutes. Subjects received verbal encouragement throughout the test and were allowed to listen to music and receive air ventilation. We protocolled cadence, actions like coughing or speaking and the exact time point of test abortion. A medical doctor reviewed the exercise and lung function data and gave the approval for participating in the study, if no contraindications based on the exclusion criteria occurred.

D Aerosol Measurement

To assess respiratory particle emission, we have built up a clean room around the exercise equipment. The base, a three-dimensional, L-formed cage, was made from a beam rail system. Around the cage, we built a triangular tent made from plastic foil to create a space of 2 m length, 2.20 m height and 1.5 m depth (Fig. S2). On one side, the tent had a flexible valve connected to a high efficiency particulate air (HEPA) filter (TAC V+; H14 filter; maximum flow rate 2200 m^3/h; Trotec™, Germany). A picture of the experimental set-up can be seen in figure S2 and S3.

The spiroergometry mask (7450 V2, Hans-Rudolph™, USA) with a Y-valve hung from the ceiling of the tent. Subjects received filtered air through a pipe connected to the valve system from their left side, to avoid contamination of the air samples. The exhaled air passed through a one-way valve into the further tube system (Fig. S3) to avoid re-breathing in the air supply. From the further tube system, the particle sample was sucked with a constant rate of 5 l/min. The particle counter (PROMO 3000 with the sensor Welas 2300; Palas™, Germany) analyzed the particle count and size distribution via scattered-light spectrometry in the size range of 0.2 to 10 µm for each second. Excess exhalation air not sampled came then to a plastic bag, to bypass inhalation time and serving as additional sampling air if $\dot{VE}$ was less than 5 l/min. Finally, a one-way outlet let excess air into the room.

We flooded the tent with filtered air at 2200 m³/h before each exercise test to ensure the air in the clean room was as particle-free as possible. Before a subject entered the clean room, two separate measurements of air quality were taken. One five-minute measurement of the filtered air coming through the spiroergometry facemask, with the goal to measure less than 30 particles/l, respectively. Then, we measured particle concentration in the clean room for another five minutes with the concentration goal of less than 150 particles/l.

We used the same bicycle ergometer and same saddle and bar settings as during the initial (CPET) exercise test. During the particle measurements, subjects were equipped with a heart rate monitor (H7; Polar Electro Oy, Finland). The protocol was identical to the initial exercise test (see section Throughout the test, we compared their cadence to the corresponding step from day one and advised them to increase or decrease their cadence if it differed. The airflow from the HEPA-filter delivered constant air ventilation at 900 m³/h. After the test, we performed two consecutive five-minute measurements of fresh air through the mask, and the clean room respectively.

Supplementary Data

In the following section data on aerosol particle concentration under constant loads of 1.9 W/kg (Fig. S4) and 3.2 W/kg (Fig. S5) (n =1) and the particle size distribution throughout the graded exercise test (n = 16) (Fig. S6) are shown. Aerosole particle concentration was measured under constant load for 25 and 30 minutes. A great variation of data can be seen across the timespan. The partcile size did not differ throughout the measurement. The particle size at rest was not significantly different than the one at maximal excerise for all participants ($p > 0.05$). No difference was found for women vs. men or trained vs. untrained subjects ($p > 0.05$).

Exercise intensity threshold 2 W/kg

By linear interpolation, we calculated the aerosol particle emission for all test subjects to uniform relative power levels in W/kg. Using these power levels, we were able to calculate mean values of aerosol particle emission. This average exceeds values of 10,000 particles/l for relative powers above about 2.1 W/kg. For reasons of practicality and considering the variation between subjects, we recommend 2 W/kg as a threshold.

Table 1. Inclusion criteria for subjects concerning Vo2 max and BMI.

**Group**	**Specific inclusion criteria**	**Specific exclusion criteria**
Endurance untrained	Self-reported VO_2max:<br>Males <55ml/min/kg<br>Females <45 ml/min/kg	BMI<19 kg/m^2<br>BMI>28 kg/m^2
Endurance trained	Self-reported VO_2max:<br>Males ≥55ml/min/kg<br>Females ≥45 ml/min/kg	BMI<19 kg/m^2<br>BMI>28 kg/m^2

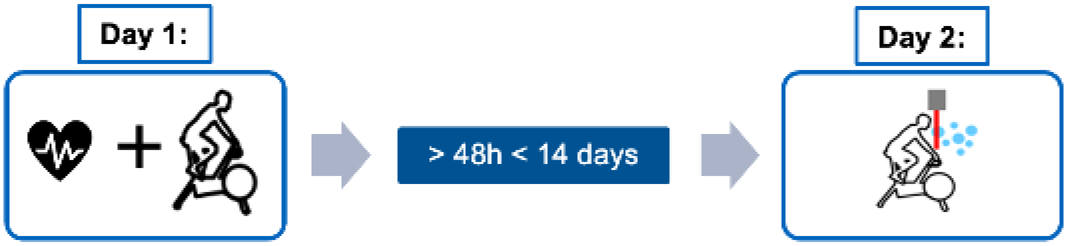

Figure S1: Study design for particle measurements during graded exercise tests on a bicycle ergometer

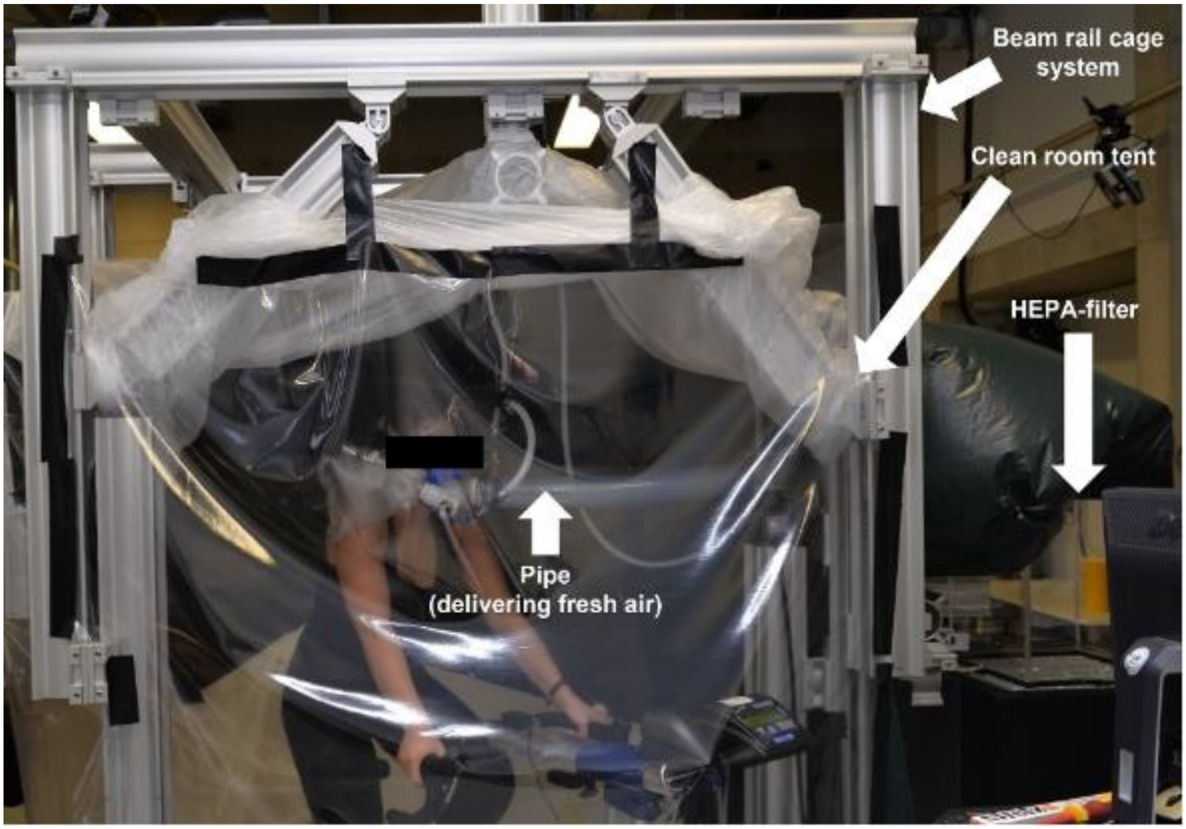

Figure S2. Set-up with clean room tent, beam rail cage, HEPA-filter and fresh air flow (tube).

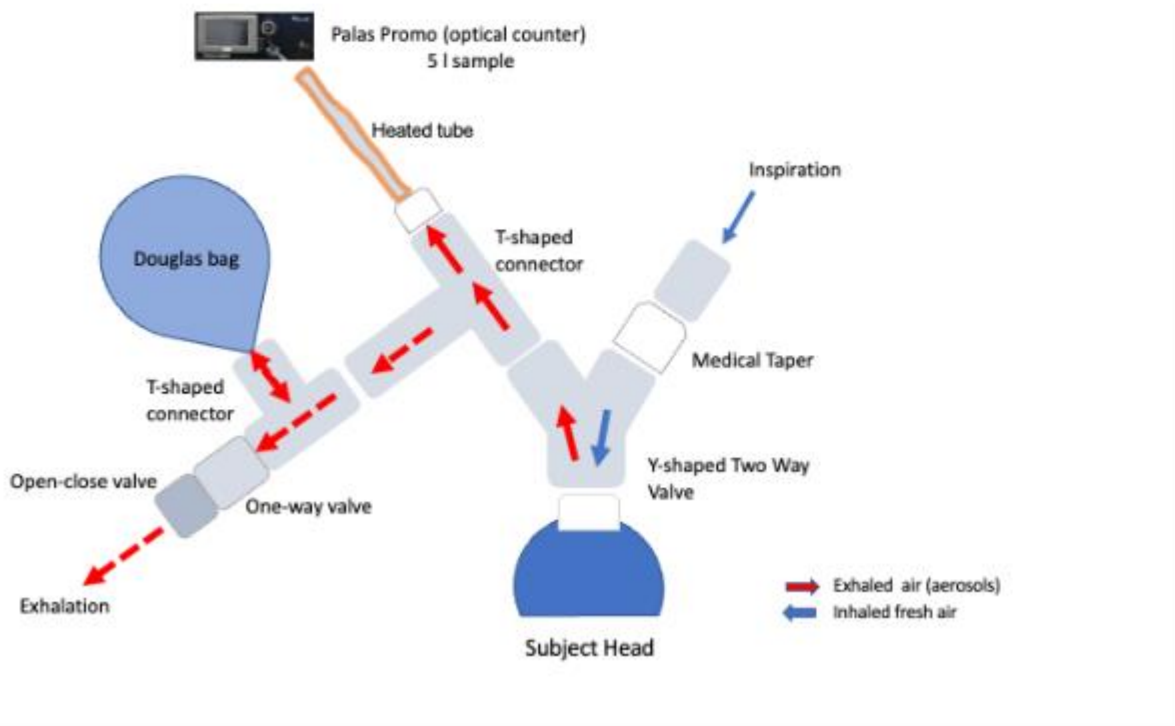

Figure S3. Schematic illustration of CPET mask with tube system and y-shaped two way valve.

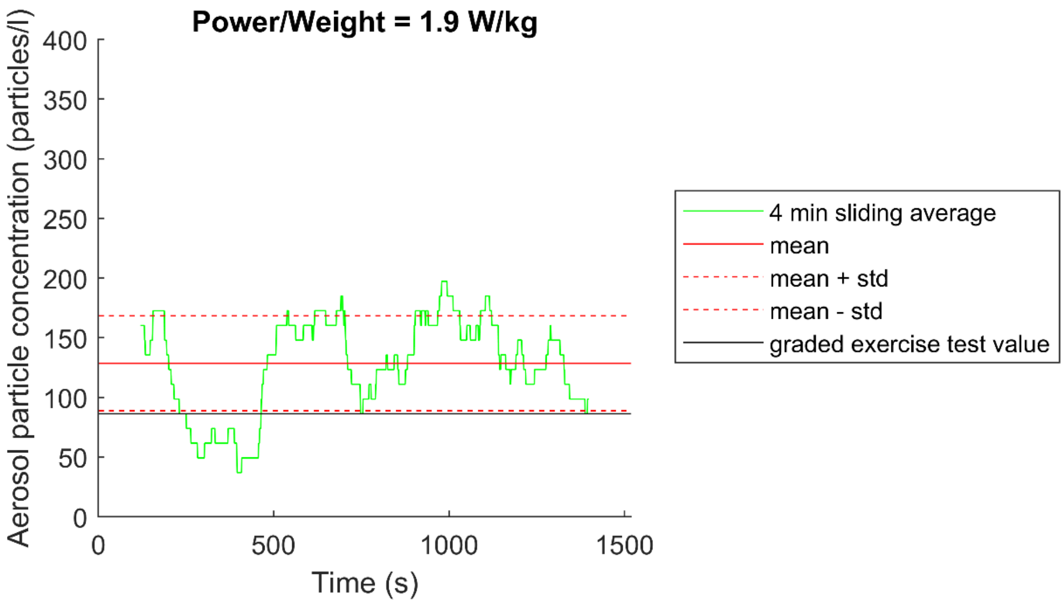

Fig. S4. Temporal aerosol particle concentration for a 25 min constant load ergometer test at 1.9 W/kg. Comparison to graded exercise test of the same subject.

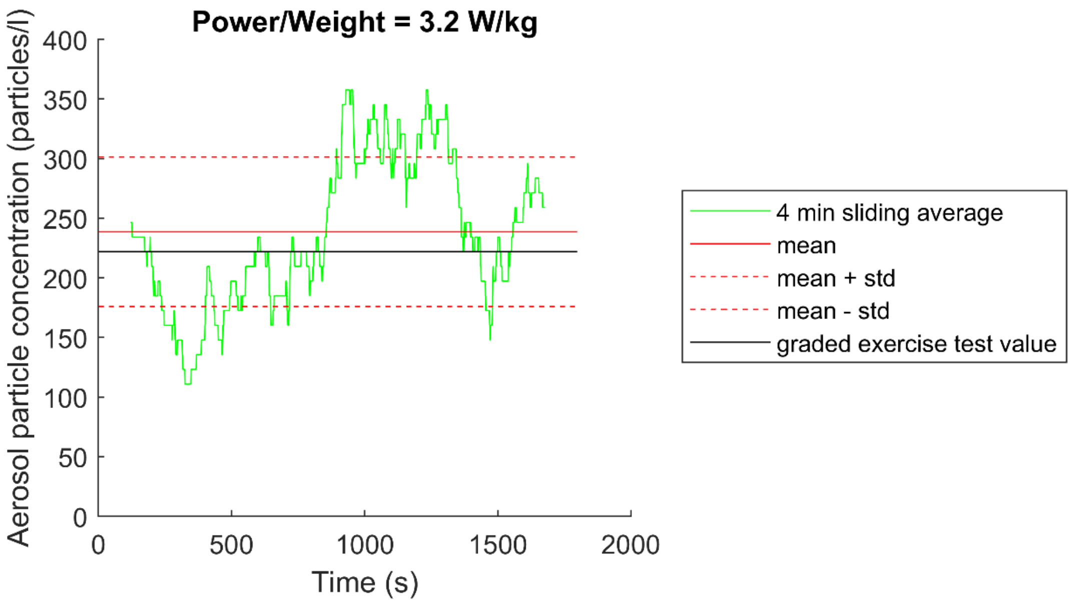

Fig. S5. Temporal aerosol particle concentration for a 30 min constant load ergometer test at 3.2 W/kg. Comparison to graded exercise test of the same subject.

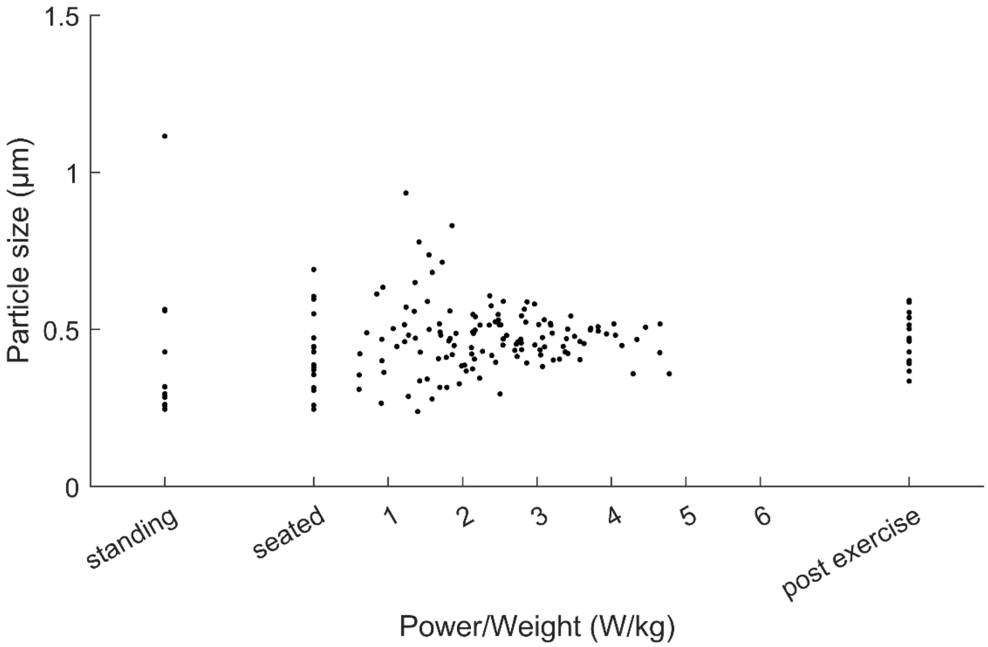

Fig. S6. Particle size distribution (assuming a refractive index of n = 1.59) for resting conditions and graded exercise until exhaustion. Mean values for 16 subjects at each intensity.

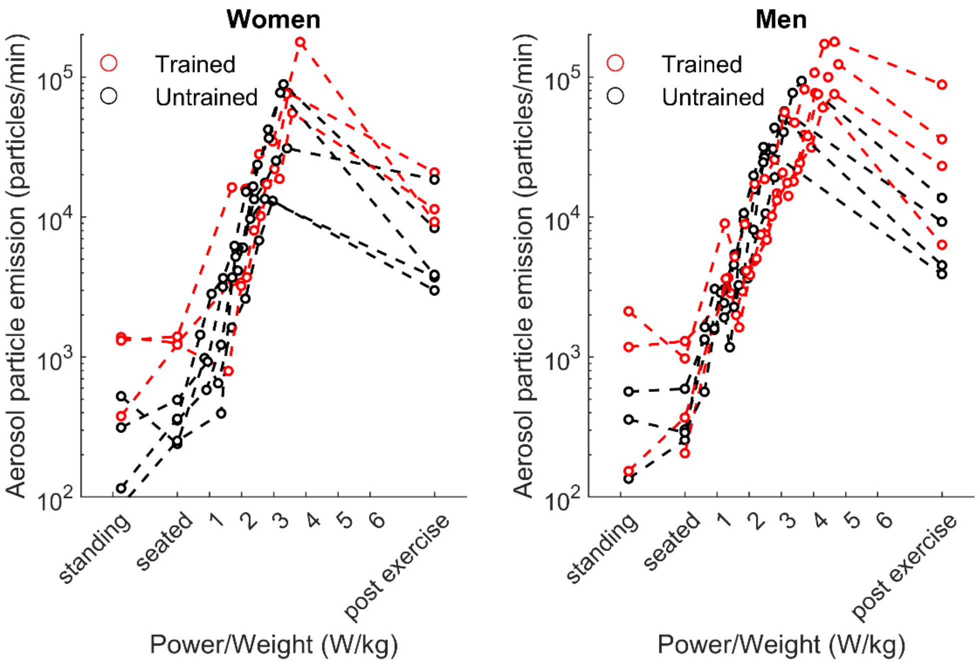

Fig. S7. Aerosol particle emission at rest and at different exercise intensities in women (n=8, left) and men (n=8, right). Standing and seated (on ergometer) values in the order of test procedure.
